# GloBIAS survey results – An insight into the global bioimage analysis community

**DOI:** 10.1111/jmi.70153

**Published:** 2026-07-27

**Authors:** Laura R. de la Ballina, Christa G. Walther, Nicholas D. Condon, Alessandro A. Felder, Martin Schätz, Bettina Schmerl, Ko Sugawara, Clara Prats, Anna Klemm, Kota Miura, Paula Sampaio, Christian Tischer, Shuichi Onami, Rocco D'Antuono, Beth A Cimini, Robert Haase, Agustin A. Corbat, Florian Levet

**Affiliations:** ^1^ Faculty of Medicine Centre for Cancer Cell Reprogramming Institute of Clinical Medicine University of Oslo Montebello Oslo Norway; ^2^ Department of Molecular Cell Biology Institute for Cancer Research The Norwegian Radium Hospital Oslo University Hospital Montebello Oslo Norway; ^3^ Project and Community Core Unit German BioImaging–Gesellschaft für Mikroskopie und Bildanalyse e.V. Konstanz Germany; ^4^ Max Perutz Labs , BioOptics ‐ Light Microscopy University of Vienna Vienna Austria; ^5^ Institute for Molecular Bioscience The University of Queensland Brisbane Queensland Australia; ^6^ Neuroinformatics Unit Sainsbury Wellcome Centre & Gatsby Computational Neuroscience Unit University College London London UK; ^7^ Viničná Microscopy Core Facility Faculty of Science Charles University Prague Czech Republic; ^8^ Department of Mathematics Informatics and Cybernetics University of Chemistry and Technology Prague Czech Republic; ^9^ Picower Institute for Learning and Memory Massachusetts Institute of Technology Cambridge Massachusetts USA; ^10^ Laboratory for Developmental Dynamics RIKEN Center for Biosystems Dynamics Research Kobe Japan; ^11^ Faculty of Health and Medical Sciences Danish BioImaging Infrastructure University of Copenhagen Copenhagen Denmark; ^12^ BioImage Informatics Unit Science for Life Laboratory and Department of Information Technology Uppsala University Uppsala Sweden; ^13^ Graduate School of Frontier Biosciences The University of Osaka Suita Osaka Japan; ^14^ i3S – Instituto de Investigação e Inovação em Saúde Universidade do Porto Porto Portugal; ^15^ Data Science Centre EMBL Heidelberg Germany; ^16^ Crick Advanced Light Microscopy STP The Francis Crick Institute London UK; ^17^ Department of Biomedical Engineering School of Biological Sciences University of Reading Reading UK; ^18^ Imaging Platform Broad Institute of MIT and Harvard Cambridge Massachusetts USA; ^19^ Center for Scalable Data Analytics and Artificial Intelligence (ScaDS.AI) Universitat Leipzig Leipzig Germany; ^20^ Facultad de Ciencias Exactas y Naturales Instituto de Física de Buenos Aires Universidad de Buenos Aires Buenos Aires Argentina; ^21^ CNRS Interdisciplinary Institute for Neuroscience University of Bordeaux Bordeaux France; ^22^ CNRS, INSERM, Bordeaux Imaging Center, BIC University of Bordeaux Bordeaux France

**Keywords:** bioimage analyst, community building, data analysis, GloBIAS, image processing, scientific society

## Abstract

There is a global need for bioimage analysis (BIA) as advances in life sciences increasingly rely on cutting‐edge imaging systems that have dramatically expanded the complexity and dimensionality of biological images. Turning these data into scientific discoveries requires scientists with effective data management skills and knowledge of state‐of‐the‐art image processing and data analysis, in other words, bioimage analysts.

The Global BioImage Analysts’ Society (GloBIAS) aims to enhance the profile of bioimage analysts as a key role in science and research. To better understand the needs and geographical representation of the BIA community, a worldwide survey was conducted, and 290 responses were collected across people from all career stages and continents. The survey underscores a strong interest of the BIA community in the activities proposed by GloBIAS to address shortcomings in work environment, funding, and scientific activities, and the enthusiasm of the community to actively contribute to the growth and sustainability of GloBIAS as a scientific society.

## 1 INTRODUCTION

Advances in bioengineering, molecular labelling and microscopy have accelerated substantially over the past 20 years, resulting in exponentially increasing volumes of image data from imaging workflows.^1^, ^2^, ^3^, ^4^ Bioimage analysis these days is an essential methodological step between image acquisition and biological interpretation, necessitating dedicated analytical expertise to enable robust and reproducible extraction of quantitative data. Practitioners in this field come from diverse backgrounds such as biology, physics and computer science, leading to huge differences in terminology, methodological expectations and professional training, therefore calling for a combined effort on creation of coherence and harmonisation across the community.

The Network of European Bioimage Analysts (NEUBIAS) established in 2016 under the EU‐funded COST Action #15124, was the first coordinated effort to formalise bioimage analysis as a professional role and aided the definition of the term ‘bioimage analyst’.^5^, ^6^ This community building approach subsequently influenced other networks including BioImaging North America (BINA), Latin America BioImaging (LABI), SingaScope and and Royal Microscopy Society (RMS).

The Global BioImage Analysts’ Society (GloBIAS) draws inspiration from these networks and aims to expand the reach to a global scale and is now an independent international non‐profit organisation, with the objective of providing a sustainable structure for community coordination, professional support and knowledge exchange in bioimage analysis irrespective of geographical origin.^7^

With the aim to assess the current status and needs of the profession at a global level, a structured community survey was conducted in 2024. In this paper, we present the survey findings, summarise the professional landscape of bioimage analysts worldwide, identify shared priorities and challenges, and outline the mission and strategic objectives of GloBIAS.

## 2 CONTENT AND ANALYSIS OF THE SURVEY

The goal of the survey was to develop a deeper understanding of the bioimage analysis community and of the individuals who identify as bioimage analysts. The survey consisted of a collection of 6 demographic questions and 22 questions addressing topics such as funding, work description, activities that would be of interest, willingness to financially or actively contribute to the society, and more (see repository link). The survey questions were developed as part of the grant 2023–321238 from the Chan Zuckerberg Initiative DAF by a subgroup of the grant applicants (the GloBIAS Steering Committee), with the help of the community manager employed on the grant. The content was based on the COBA survey question list (https://doi.org/10.1111/jmi.13229) and it has been modified to inform our priorities and collaborative efforts for the Global BioImage Analysts’ Society. The survey contained 25 multiple choice and 3 short answer questions, all of which were optional. Please note that at the time of the survey, GloBIAS as an association did not exist yet. The questions were further divided into four sections: Intro and consent to data being used in future publications while remaining anonymous, Demographic questions (1 to 6), Community group and needs (Q1 to Q9), How you would like GloBIAS (as a non‐profit association) to be organised: activities and interaction opportunities (Q10 to Q22).

The survey was open between February 7 and April 29 2024 as a Google Form. It was distributed via social media (X, LinkedIn of GloBIAS and its supporters via reposting), via emailing across the different imaging community newsletters (e.g. NEUBIAS, BioImaging North America, Royal Microscopical Society, BioImaging UK, Light Microscopy Australia, Latin America BioImaging, African BioImaging Consortium) list servers (e.g. Confocal list server, Image.sc) and contacts within professional networks of GloBIAS members were used to invite more participants to the survey.

The survey results are accessible and can be explored in a marimo dashboard available at: https://globias‐bioimageanalysts.github.io/Survey_2024, thanks to the consent granted by all the responders to publish their data. The analysis was performed by the GloBIAS Survey 2024 Work Group composed of at least 7 coders among the authors using Python via Jupyter notebooks,^8^ to be able to keep track of the analysis. The replies were exported in a CSV file and later processed with Pandas^9^ library. In particular, the data were preprocessed by removing or replacing special characters in questions and answers (as described in the source cleaning notebook) and one reply was removed as only demographic information was filled in. The preprocessed file containing the replies was then analysed in several different notebooks addressing different questions and cross‐analyses as it can be seen in the notebook folder of the GitHub repository. Some respondents did not answer all the questions leaving missing data. In these cases, analysis was performed only with the remaining answers.

Only career stage related questions and Q9, which was free‐text response, were further processed, while most of the questions were analysed as they were. In particular, the positions held by respondents were grouped as: *junior*, if they corresponded to a position prior to and not including postdoctoral position; *mid‐career* included postdocs to junior group leaders and assistant professors; while *leaders* comprised associate professors, facility heads, principal scientists and directors; finally, staff positions typically contained terms such as engineer, scientist, researcher, specialist, staff and no indication of seniority (see notebook). Additionally, Q9 asked ‘How can GloBIAS support you in your bioimage analysis endeavours?’, as an open question. In this case, all answers were read by two separate GloBIAS members and added to whether the answer addressed the following topics: Training and Education, Networking and Community, Conferences, Technical Support, Infrastructure, Best practices, Sponsoring, and Outreach. Some unique answers were also highlighted. As the number of responses when grouped into categories, such as grouping by continent, were considered low to perform statistical analysis, we refrained from doing so.

## 3 RESULTS

### 3.1 Demographics

The distribution of demographics showed that while most of the 290 respondents were based in Europe (178 respondents; 61%), all geographical regions were represented (see Figure 1). With regard to gender, 164 (57%) identified as male, 114 (39%) as female, with a small number selecting ‘prefer not to say’, leaving it blank, or selecting ‘other’ (12 respondents; 4%). Female to male ratio (calculated as percentage of females divided by percentage of males without taking into account the remaining categories) per continent was between 0.65 and 1.46 for every continent which, taking into account the number of responses, did not translate into a significant bias. About 60% (176 of 289 responses) of respondents had a permanent position (see Figure 1; 64% for male, 56% for female). Most of the respondents (90%) were working in an academic environment, while the remaining respondents worked in a for‐profit entity, and no regional differences were apparent when grouping by continent. Additionally, most of the respondents had staff positions (41%) followed by a leadership (24%) or mid‐career role (19%). Together, these data highlight a predominantly Europe‐based, academic respondent cohort working across diverse roles and career stages. Collectively, taking part in the survey already shows interest in connecting with other bioimage analysts worldwide.

**FIGURE 1:**
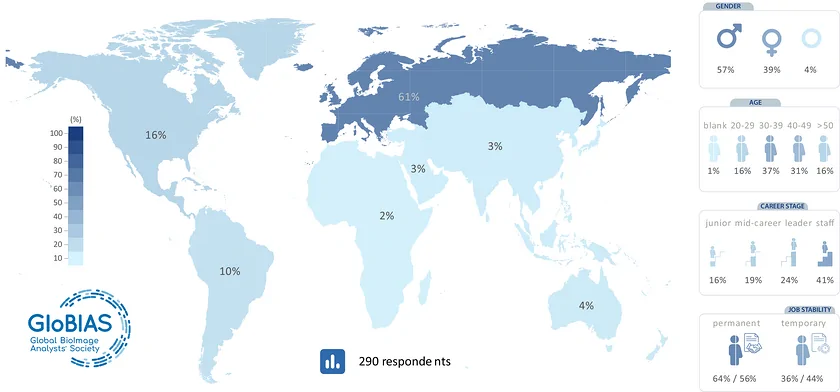
Demographics of the survey responses. Map representing the percentages of the 290 responses coming from people working in each geographical region. Specifically, there were 178 (61%) from Europe, 47 (16%) from North America, 28 (10%) from South America, 12 (4%) from Australia/Oceania, 10 (3%) from Asia, 8 (3%) from Near/Middle East, and 6 (2%) from Africa. With regard to gender, there were 164 (57%) male respondents, 114 (39%) female respondents, and 12 (4%) respondents who replied ‘prefer not to say’, ‘other’ or gave no response. Note that questions could be left blank, so answers may not sum to 100%. With regard to age groups, there were 46 (16%) of respondents between 20 and 29, 106 (37%) between 30 and 39, 89 (31%) between 40 and 49, and 47 (16%) above 50 years old. After classifying the positions into career stages, 46 (16%) of respondents had a junior role, 55 (19%) had a mid‐career level, 69 (24%) had a leader role, and 120 (41%) had a staff position. Finally, 174 (60%) respondents have a permanent position, in particular 64% for male and 56% for female.

### 3.2 Duties and cost recovery model for bioimage analysts

We aimed at identifying the tasks that are part of the daily work of our community. From the responses to the GloBIAS survey it emerges that the role of the bioimage analysts includes a range of tasks such as instruction, service, leadership of own projects, management, tool development, and infrastructure maintenance (Figure 2). Almost 90% (208 out of 236) of the respondents spend time on instruction, highlighting this as the most common task bioimage analysts devote time to. More than 138 of the respondents also work on either their own projects (159), delivering a service (151), or in management and administration (138). Finally, less than 116 of the respondents allocate time on tool development (112) and infrastructure maintenance (115).

**FIGURE 2:**
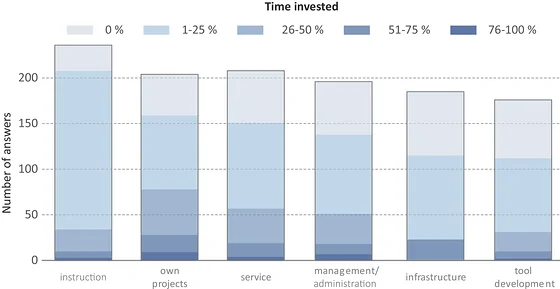
Time spent by survey respondents in different duties. Stacked bar plot showing the percentage of time invested in different tasks by survey respondents answering the question (total number of responses per question: instruction (236), own projects (204), service (208), management/administration (196), tool development (176) and infrastructure (185)).

A critical determinant for what bioimage analysts spend their time doing is the funding model of the lab or facility where they work. We therefore set out to quantify the funding sources primarily funding bioimage analysis activities in core facilities, where funding is likely to be more variable; respondents who work in facilities were asked to indicate the support percentages (in increments of 25%) for their facility for various categories, while respondents who did not work in facilities were asked not to respond. 155 of 290 respondents entered nonzero values for at least one funding category of their own grants, others' grants, internal support, microscope fees, recharge from analysis projects, consulting fees, or that they weren't sure. Internal funding made up the largest fraction of known funding sources (reported in 105/155 entries; highest funding or tied for highest funding in 75/155 entries), though as previously reported,^10^ fewer than 20% of responses indicated 75%–100% funding from these sources. Of the remaining sources, microscope fees were the next most prevalent (83 reports; highest funding/tied for highest funding in 52 entries), followed by others' grants (79 reports, 27 (tied)‐majority), own grants (61 reports, 21 (tied)‐majority), analysis project fees (49 reports, 18 (tied)‐majority), and consulting fees (25 reports, 8 (tied)‐majority) (Figures 3 and S1). Overall, after applying a weighted averaging approach to account for ties and reports of less or more than 100% (due to imprecision or the large bins of our metric), 30% of community funding for bioimage analysts working in facilities comes from institutional support, 20% from microscope fees, 15% from others' grants, 11% from own grants, 9% from analysis project fees, and 4% from consulting fees, with 11% unknown. The large difference between reported total budget percentages for microscope fees versus image analysis recharge fees represents an interesting data point for future investigation; at its most benign interpretation, it could merely represent that many bioimage analysts are embedded in microscopy facilities, but may also indicate that in some or many cases that microscope recharge fees are being used to subsidise user unwillingness to pay the full costs of analysis work, which has been previously reported for other computational facilities in academia.^11^ To see if certain facility ‘archetypes’ could be discerned in the data, the responses were turned into a similarity matrix and hierarchically clustered (see Figure S1). More than 60% of responses fell into a cluster dominated by a mixture of high internal support and/or microscope fee support, with other clusters for other models where funding percentages were known representing <10% of responses each.

**FIGURE 3:**
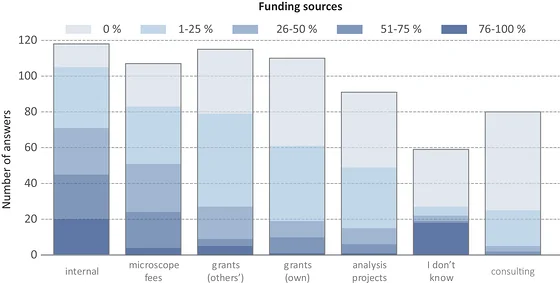
Funding sources in core facilities. Of known funding sources, internal funding and microscope fees make up the largest budget fractions for those doing bioimage analysis in a facility; in decreasing order of reported fractions, others' grants, own grants, analysis projects, and consulting fees are also used to fund bioimage analysis in facilities (total number of responses per question: internal (118), microscope fees (107), other's grants (115), own grants (110), analysis projects (91), I don't know (59) and consulting (80)).

### 3.3 Interests of the bioimage analysis community

With regard to the interests of the community, the responses (144 responses) demonstrate a strong interest in training and skill development, with a desire for learning opportunities ranging from introductory to advanced levels, and covering specific areas such as statistics, machine learning and deep learning. Networking and community building are also highly valued, highlighting the importance of connection, collaboration, knowledge exchange and communication. Conferences are recognised as valuable opportunities for training, networking and knowledge exchange. Technical support is a significant concern, with the community seeking guidance and assistance from experts, particularly for project‐specific questions and data management. Furthermore, the community requires robust infrastructures to support their activities, and is committed to best practices, standards and open‐source methodologies. Finally, outreach and sponsoring are considered important to promote accessibility and facilitate participation in events and training programmes (see Figure 4).

**FIGURE 4:**
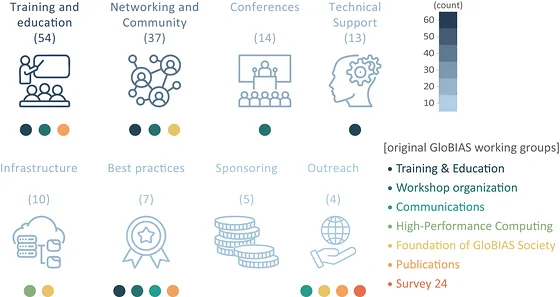
Number of respondents showing interest in different topics and their alignment with GloBIAS' working groups. Icons are colour‐coded by the number of survey free‐text responses expressing interest in activities related to Training and Education (54), Networking and Community (37), Conferences (14), Technical Support (13), Infrastructure (10), Best practices (7), Sponsoring (5), and Outreach (4). Below each topic the original GloBIAS working groups developing activities related to the aforementioned interests are indicated.

Beside the interests of respondents in specific workgroups, some unique responses worth highlighting are collaborative research, tool development, hackathons, career development, data management, quality control, and financial support. Specific technical gaps were also identified, such as a request for training on basic algorithms and whole‐slide imaging which was also raised during the 2025 GloBIAS conference in Kobe, Japan.

Regarding future GloBIAS activities, 97% of respondents (276 of 285) prioritised online events, with ‘new bioimage analysis tools’ emerging as the most popular topic. In‐person workshops on new tools also garnered significant interest (90%; 260 of 281), alongside sessions on infrastructure, image data management, and the exploration of open questions and datasets (Figure 5). In terms of frequency, the majority of participants preferred online events to be held on a quarterly basis.

**FIGURE 5:**
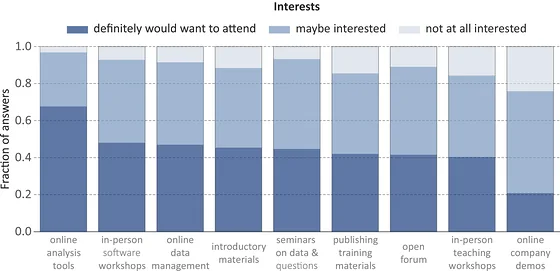
Interests in different bioimage analysis activities. Stacked bar plot showing the fraction of survey respondents who indicated their interest level in the different proposed activities (total number of survey respondents per column was: online analysis tools (285), in person software workshops (281), online data management (283), introductory materials (284), seminars on data & questions (282), publishing training materials (284), open forum (284), in‐person teaching workshops (275) and online company demos (278)).

Amongst the responses, several unique and interesting suggestions emerged for potential events in the field. Notably, there are calls for teaching good practices and for offering introductory bioimage analysis courses, both of which could be highly beneficial for the bioimage analysis community. Additionally, quality control and fraud prevention were highlighted as important areas to address. In‐person events focusing on image data infrastructure and management, as well as the formation of a core facilities group, were also mentioned. Respondents also expressed interest in organising ‘meet and greet’ retreats for researchers to foster connections. Furthermore, the idea of creating a bioimage analysis journal was proposed.

The GloBIAS related grant proposal already addresses many of the community interests (Figure 5 and individual responses), and new areas have been identified through the survey, allowing GloBIAS to shape its activities according to the needs of the community. Previous activities of NEUBIAS such as curating a software tools catalogue (biii.eu), covering the fundamentals of bioimaging (https://www.bioimagingguide.org/), and creating easy‐to‐use tools to develop and deploy workflows (such as Galaxy, NextFlow or MCMICRO) were also mentioned in the individual responses.

These articulated interests span the essential skills and knowledge for the future development of bioimage analysis and can only be fully addressed through the involvement of a broad and active community.

### 3.4 Willingness of the respondents to actively contribute to GloBIAS

Respondents from all over the globe expressed their willingness to actively contribute in society activities as shown in Figure 6 (157 out of 278). Although the overall low number of responses in some regions is concerning, this probably does not reflect the overall interest in bioimage analysis in those regions but the role of bioimage analyst being barely established.

**FIGURE 6:**
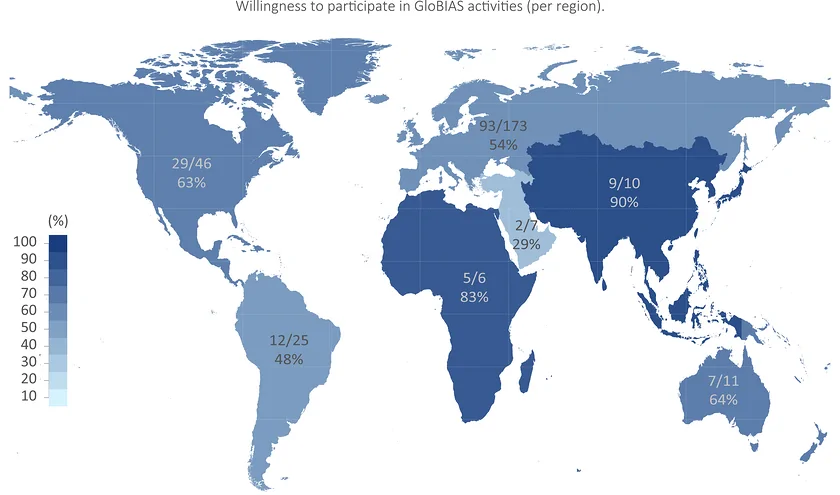
World map depicting the willingness to actively contribute to GloBIAS’ activities. Analysing the responses to the GloBIAS survey 2024 from each different region, as follows: Europe, 93 out of 173 (54%); North America, 29 out of 46 (63%); South America, 12 out of 25 (48%); Australia/Oceania, 7 out of 11 (64%); Asia, 9 out of 10 (90%); Africa, 5 out of 6 (83%); and Near/Middle East, 2 out of 7 (29%).

In order to support the needs and interests of the bioimage analysis community, and to achieve the goals and mission of the GloBIAS initiative, the survey included questions to probe the willingness and scope of community contribution in GloBIAS activities. Overall, 56% (155 of 274) of the respondents indicated their willingness in helping to organise and run regular events. Additionally, when asked in which GloBIAS WG the survey participants would like to join, 65.5% of the respondents (190 of total 290) selected at least one, often multiple WG. More than 50 volunteers at the time of starting the analysis of this survey joined one or two WG (see GloBIAS working groups webpage^1^) and the GloBIAS Association has more than 80 members. Reflecting the community's priorities, the *Training & Education* and *Workshop 2024* WG, tasked with organisation and programming of the first annual workshop in November 2024 in Gothenburg, garnered the most significant initial membership, with over 10 members each. Survey participants indicated equal interest in contributing to WG hosting training events and creating the material, as well as establishing criteria for scientific data integrity in bioimage analysis (104 responses each). Slightly fewer indicated interest in WG to prepare online events and annual meetings (90 responses) or in‐person events and hackathons (80 responses). Finally, 57 respondents expressed willingness to engage in outreach activities of the society, such as social media channels and conference presentations. When queried about factors that would encourage further contribution, the majority of bioimage analysts cited training opportunities as key. Open communication channels, online events, and alignment with scientific expertise were also frequently mentioned, with an open help desk noted by many.

These results underscore the bioimage analysis community's enthusiasm for collaboration and collective growth, enabling GloBIAS to support evolving global BIA needs through targeted training, accessible communication, and inclusive opportunities for contribution.

## 4 DISCUSSION

In this article, we report survey data collected in 2024 from the global bioimage analyst community. Respondents represented a range of career stages and were geographically diverse, with the majority identifying as male. While participation spanned multiple regions, a substantial portion of responses came from individuals based in the Global North, reflecting the historical development of the field, initially concentrated in Europe and North America, and more recently expanding into South America, Asia and Africa. This uneven distribution was likely sparked by historical reasons and underscores the importance of continued outreach, and GloBIAS has made significant efforts in this direction. Notably, the 2024 workshop in Gothenburg included invited participants from underrepresented regions, and the 2025 conference in Kobe further strengthened ties with the Asian bioimage analysis community and promoted mutual knowledge exchange. Given the limited sample size and inherent biases, our interpretation focuses on identifying shared challenges and community priorities rather than drawing firm quantitative conclusions.

Respondents undertake a diverse set of daily responsibilities that span technical, managerial and service tasks. While domain‐specific expertise is required, many of these tasks are also found in traditional academic roles (own projects, instruction, management) and more generalist non‐traditional technical roles such as research software engineers (tool development, infrastructure, service) and imaging scientists (service, instruction). This highlights the importance of bioimage analysts interacting with adjacent communities, as well as the fluid boundaries between roles.^12^, ^13^ The survey responses confirm that the GloBIAS objective to enhance and harmonise the bioimage analysts role in science and research fills a critical void that individual practitioners have long navigated alone, with those reporting diverse daily duties.

The majority of respondents expressed interest in training‐ and skills‐development‐related activities and materials. This strong interest is directly mirrored in the high initial membership and activity within the GloBIAS Training & Education Working Group. Beyond technical skills, networking, collaboration, and knowledge exchange are highly valued, with conferences recognised as crucial venues for these activities. Furthermore, a strong commitment to adopting best practices, community standards, and open‐source methodologies emerged as a shared priority.

Crucially, the continuation of GloBIAS is well supported as 56% (157 out of 278) of respondents indicate a willingness to participate in organising and running events and 72% (201 out of 278) mentioned willingness to pay a fee to become a member of the society. This enthusiasm is reflected in the significant number of volunteers joining working groups, particularly those focused on training and education and annual workshop organisation. Moving forward, the society must address systemic challenges related to funding and institutional support to effectively leverage this collective commitment and ensure the longevity of BIA as a key scientific role.

## AUTHOR CONTRIBUTIONS

**Conceptualisation**: CG Walther, C Prats, F Levet, A Klemm, R D'Antuono, BA Cimini, R Haase. **Data curation**: CG Walther, AA Felder, BA Cimini, R Haase. **Analysis**: AA Corbat, CG Walther, ND Condon, AA Felder, M Schätz, B Schmerl, BA Cimini. **Funding acquisition**: C Prats, F Levet, P Sampaio, C Tischer, R D'Antuono, BA Cimini, R Haase. **Methodology**: AA Felder, F Levet, BA Cimini, R Haase. **Project administration**: CG Walther, C Prats, C Tischer. **Software**: AA Corbat, AA Felder, M Schätz, BA Cimini. **Supervision**: AA Corbat, CG Walther, C Tischer, R D'Antuono, R Haase. **Validation**: AA Corbat, K Sugawara. **Visualisation**: AA Corbat, LR de la Ballina, ND Condon. **Writing – original draft**: AA Corbat, CG Walther, LR de la Ballina, ND Condon, AA Felder, M Schätz, B Schmerl, F Levet, R D'Antuono, R Haase. **Writing – review & editing**: AA Corbat, CG Walther, LR de la Ballina, ND Condon, AA Felder, M Schätz, B Schmerl, K Sugawara, C Prats, F Levet, A Klemm, K Miura, P Sampaio, C Tischer, S Onami, R D'Antuono, BA Cimini, R Haase.

## CONFLICT OF INTEREST STATEMENT

K.S. is employed part‐time by LPIXEL Inc. All other authors do not declare any conflicts of interest.

## FUNDING

The following grants from the Chan Zuckerberg Initiative DAF, an advised fund of Silicon Valley Community Foundation, are reported: N.D.C. was supported as a CZI Imaging Scientist by grant number 2023–329684, A.F. was funded by grants 002022–309537 and 2024–349556, B.A.C. was supported by 2023–329649, C.G.W. by 2023–321238 to R.H., for the foundation of GloBIAS (hosted by German BioImaging); C.T. by 2020–225265. B.A.C. was additionally supported by NIH P41 GM135019. L.R.B was supported by the Research Council of Norway through FRIPRO grant number 314684. B.S. is funded by DFG project 524025372 and additionally supported by NIH subaward RM1NS132981, and ONR grant N000142412055 to Elly Nedivi, and the Picower Institute Innovation Fund. This work was supported by the Francis Crick Institute, which receives its core funding from Cancer Research UK (CC1069), the UK Medical Research Council (CC1069) and the Wellcome Trust (CC1069). This work was supported by Viničná Microscopy Core Facility (RRID:SCR_026602) co‐financed by the Czech‐BioImaging large RI project LM2023050. The SciLifeLab BioImage Informatics Unit (A.C. and A.K.), a unit of the National Bioinformatics Infrastructure Sweden, receives funding from SciLifeLab and the Swedish Research Council (VR). P.S. was supported by grant FCT/EULAC/0002/2022. F.L. was supported by the Ministère de l'Enseignement Supérieur et de la Recherche (ANR‐20‐CE11‐0006 NANO‐SYNATLAS). S.O. was supported by JST CREST (JPMJCR1926); JST NBDC (JPMJND2201).

## DATA AND CODE AVAILABILITY STATEMENT

The data and the code used to analyse the survey responses are distributed with the CC BY 4.0 License and are available at: https://github.com/GloBIAS‐BioimageAnalysts/Survey_2024. The survey data can also be explored in a dashboard available at: https://globias‐bioimageanalysts.github.io/Survey_2024/.

## Supporting information



Supporting Information

Supporting Information

## Footnotes

1
https://www.globias.org/activities/working‐groups
